# An ultrasensitive planar array p24 Gag ELISA to detect HIV-1 in diverse biological matrixes

**DOI:** 10.1038/s41598-021-03072-7

**Published:** 2021-12-08

**Authors:** Callie Levinger, J. Natalie Howard, Jie Cheng, Pingtao Tang, Amit Joshi, Marta Catalfamo, Alberto Bosque

**Affiliations:** 1 Department of Microbiology, Immunology and Tropical Medicine, George Washington University, Washington, USA; 2 Quanterix Corporation, Billerica, MA USA; 3 Department of Microbiology and Immunology, Georgetown University School of Medicine, Georgetown University, Washington, USA

**Keywords:** Microbiology, Diseases

## Abstract

Human immunodeficiency virus-1 (HIV-1) persistence in the presence of antiretroviral therapy (ART) has halted the development of curative strategies. Measuring HIV persistence is complex due to the low frequency of cells containing virus in vivo. Most of the commercially available assays to date measure nucleic acid. These assays have the advantage of being highly sensitive and allow for the analysis of sequence diversity, intactness of the HIV genome or evaluation of diverse RNA species. However, these assays are limited in evaluating translational competent viral reservoirs. In here, we developed an ultrasensitive p24 ELISA that uses the Simoa planar array technology that can detect HIV-1 virions and HIV-1 infected cells with limit of detection similar to nucleic acid assays. Furthermore, the assay is optimized to measure very low levels of p24 in different biological fluids without a major loss of sensitivity or reproducibility. Our results demonstrate that the ‘homebrew’ planar p24 ELISA immunoassay is a broadly applicable new tool to evaluate HIV persistence in diverse biological fluids and cells.

## Introduction

Human immunodeficiency virus-1 (HIV-1) persistence is responsible for the increase in viremia observed after antiretroviral (ART) interruption and can be a driver of residual immune activation observed in (ART)-treated people living with HIV (PLHW)^1^. Several interventions are currently under investigation to reduce, eliminate or permanently silence this latent reservoir^2–7^. To date, several assays have been developed to assess HIV-1 persistence (For a review^8^). The development of ultrasensitive and reproducible assays is warranted to evaluate the clinical efficacy of cure strategies. Furthermore, the development of reliable assays that can detect HIV directly in different biological matrixes will aid in our understanding of HIV persistence in different anatomical compartments. Most of the current assays to evaluate persistence measure HIV-1 DNA or RNA. These assays are really effective to evaluate HIV-1 persistence and allow for the analysis of integrated virus, sequence diversity, intactness of the HIV genome or evaluation of diverse RNA species, including splicing forms or poly-A mRNA^9–13^. However, these assays are limited in evaluating translational competent viral reservoirs. Assays to evaluate translational competent viruses have been lagging behind due to the lack of protein-based assays with limit of detection (LOD) similar to that of nucleic acid-based ones. Flow cytometry assays to detect cells harboring translational competent viruses have been developed but require a large number of cells due to the lack of sensitivity and high background of flow cytometry^14,15^. To our knowledge, only one enzyme-linked immunosorbent assay (ELISA)-based assay has been developed so far that measures HIV Gag protein expression with the sensitivity of nucleic acid-based assays using a digital ELISA (dELISA) platform^16–22^. The dELISA recognizes HIV-1 protein at the low fg/ml equal to 50 HIV RNA copies/ml in serum or plasma^16,17^. However, it requires a high volume of sample (300 μl to run in duplicate) and needs further optimization for quantitation in different biological matrixes.

In this work, we have evaluated a homebrew p24 immunoassay developed using the ultrasensitive Quanterix Simoa planar array technology and the SP-X imaging and analysis system^23^. The advantages of this new platform are that: (1) it can be performed using volumes between 25 to 50 μl in a 96-well format; (2) it can be optimized and developed to the protein of interest; (3) it allows for the direct quantification in different matrixes without further manipulation; and (4) the full assay can be performed in less than 5 hour. We demonstrate that this assay has a LOD in the low fg/ml using only 50 μl of sample. Furthermore, we have assessed the ability of this assay to measure HIV-1 p24 directly in different tissue culture media; biological fluids including human plasma, human serum (huSerum), cerebrospinal fluid (CSF) and breast milk; and cell lysates. Finally, we validated the assay using cells isolated from PLWH. Our results indicate that the assay is highly reproducible and could potentially detect as low as a single viral particle in certain matrices and a single infected cell in cell lysates. As such, the homebrew planar SP-X immunoassay p24 ELISA can be an additional assay to evaluate HIV-1 cure approaches when sample volume is limited, and to study HIV-1 persistence in diverse anatomical compartments.

## Results

### A homebrew p24 ELISA with extended range of quantification

**Figure 1:**
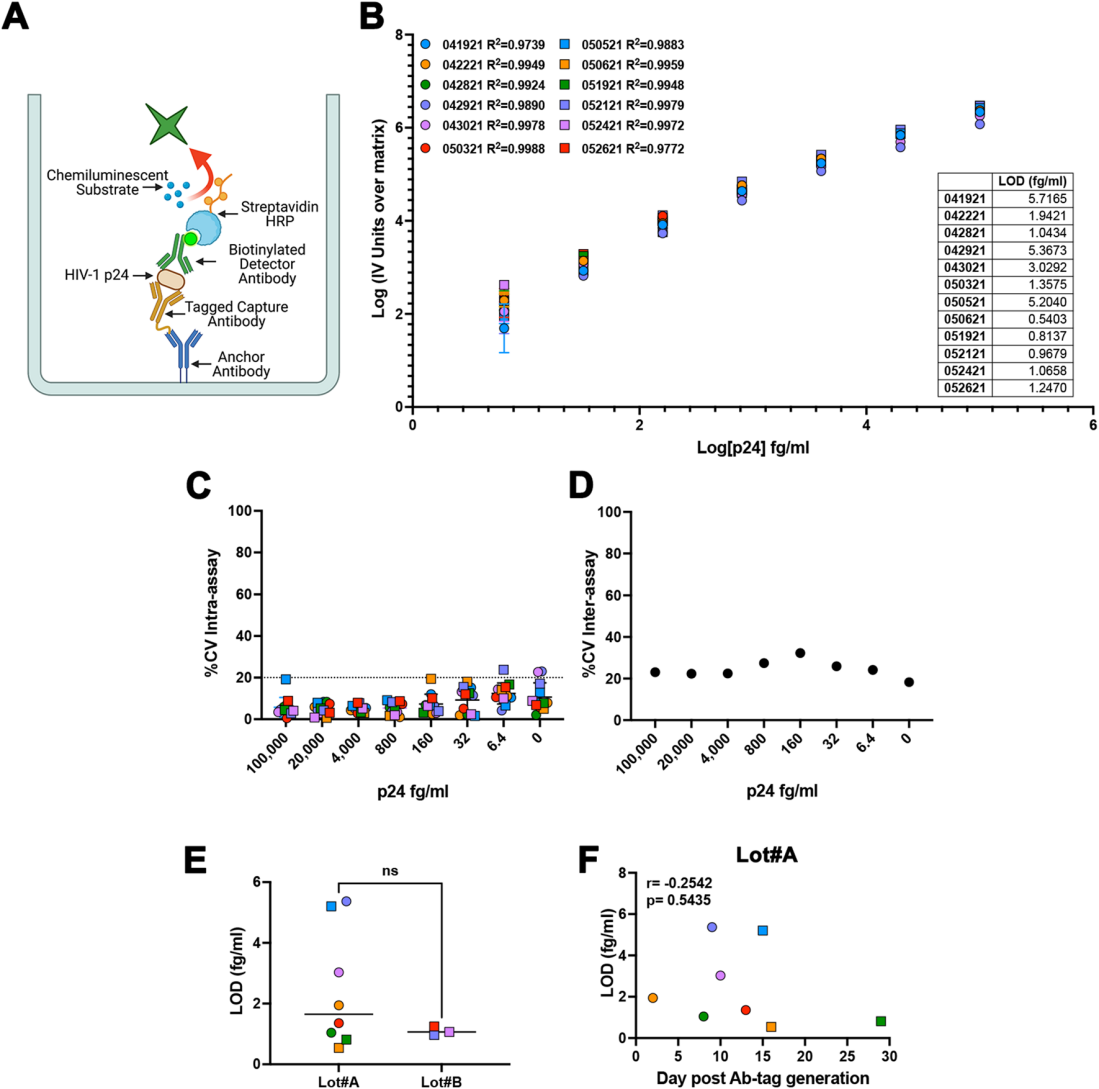
Detection of single viral HIV-1 particle using the homebrew Simoa planar array p24 ELISA. (**A**) Simoa Planar Homebrew overview. Created with Biorender.com. (**B**) Standard curves of 12 independent experiments using a range of concentrations from 100 pg/ml to 6.4 fg/ml of p24 prepared in homebrew sample diluent. Each standard was done in technical triplicates. Data has been transformed by subtracting the IV units from the matrix (0) and calculating the log10 from both the IV units and the p24 concentration. Low limit of detection (LOD) for each experiment is provided as table. (**C**) % of the coefficient of variation (%CV) intra-assay for each concentration for the 12 independent experiments. (**D**) % of the coefficient of variation (%CV) inter-assay for each concentration. (**E**) Capture and detector antibody pair batch effect on the LOD. (**F**) Correlation of the time after capture and detector antibody pair preparation for Lot#A and the LOD, calculated using Pearson correlation.

The homebrew Simoa planar ELISA immunoassay uses an anchor antibody microprinted in microwell plates specific for a peptide tag (Fig. 1A). The peptide tag is then conjugated to an antigen specific capture antibody via maleimide chemistry, enabling high affinity binding of capture antibody to the anchor antibody. Samples are incubated directly and bound antigen is sandwiched between the peptide-tagged capture and biotinylated detector antibodies. Next, streptavidin horseradish peroxidase (HRP) is added, followed by a final wash step. Then, the plate is pat dried and a mixture of chemiluminescent substrate Luminol and peroxide is added to the wells. The plate is scanned using Quanterix SP-X imager to measure the enhanced Chemiluminescence signal generated from the enzymatic reaction of HRP with hydrogen peroxide and Luminol (Fig. 1A). Using this methodology, we have developed a homebrew Simoa planar HIV-1 p24 ELISA. First, we evaluated the assay using the HIV-1 p24 antigen standard (heat-inactivated viral antigen from HIV-1IIIB) from HIV-1 p24 Antigen ELISA 2.0 (ZeptoMetrix Corporation) prepared in Simoa Planar Array Homebrew Diluent A (sample diluent). The dynamic range of the planar array immunoassay is from 2 ng/ml to 128 fg/ml with a limit of detection (LOD) of 35 fg/ml, extending the range of detection from other commercial ELISAs and allowing to measure levels of HIV-1 p24 protein in the fg/ml range (Extended Data Fig. 1). Furthermore, this assay has an excellent regression line fit (R^2^), low coefficient of variability (%CV) and good recovery (%RE) (Extended Data Fig. 1C-D).

### Evaluation of the sensitivity and reproducibility of the homebrew Simoa planar p24 ELISA immunoassay

To further evaluate the sensitivity and reproducibility of the assay, we performed a series of 12 biological replicates spiking p24 protein in sample diluent in a range of concentrations spanning from 100 pg/ml to 6.4 fg/ml. Each concentration and the blank control were done in technical triplicates. As shown in Fig. 1B, the assay had excellent R^2^ at these lower concentrations. It has been estimated that one picogram of p24 protein equals 10^4^ virus particles or 10 viral particles per fg^24^. The LOD of the assay is between 0.5 to 5 fg/ml or 5 to 50 viral particles/ml, similar to the LOD of the previous dELISA. The average LOD between the 12 replicates is 2.36 ± 1.96 fg/ml or 23.6 ± 19.6 viral particles per ml suggesting that the assay could potentially measure as low as 1.18 ± 0.98 viral particles in 50 μl of assay diluent.

Next, we addressed the intra and inter assay variability to evaluate the reproducibility of the assay. The average %CV is below 20% for all of the different standard concentrations including the matrix alone (Fig. 1C). The assay did have a higher inter-assay %CV of approximately 20%. These results suggest that the assay was highly reproducible within an individual plate, but has a higher inter-assay variation. This inter-assay CVs could be improved with automated wash steps. This assay relies on the generation of a pair of tagged capture and detection antibodies (Fig. 1). We first wanted to evaluate whether the sensitivity of the assay depends on antibody batch. As shown of Fig. 1E, there was no statistically significant difference between the two batches used. The tagged capture and detection antibodies can be stored at 4 °C upon generation. We then evaluated the stability of the antibodies and found that the antibodies can perform to a similar degree for up to 30 days without losing sensitivity (Fig. 1F).

In conclusion, we demonstrated that the homebrew Simoa planar p24 ELISA is a highly reproducible assay with a limit of detection similar to the previous digital ELISA. This new assay has low intra-assay variability regardless of antibody batch and stability of the tagged antibodies for of up to 30 days.

### Evaluation of the homebrew Simoa planar p24 ELISA immunoassay in diverse matrixes

Based on the high sensitivity and reproducibility of the planar array assay in the sample diluent, we wanted to evaluate its performance in more complex matrixes. Similar to the sample diluent matrix, we performed a series of 3 biological replicates using a range of concentrations spanning from 100 pg/ml to 6.4 fg/ml in triplicates and a blank control in different commercially available biological fluids from HIV-1 negative donors, cell growth media, and plasma.

**Figure 2:**
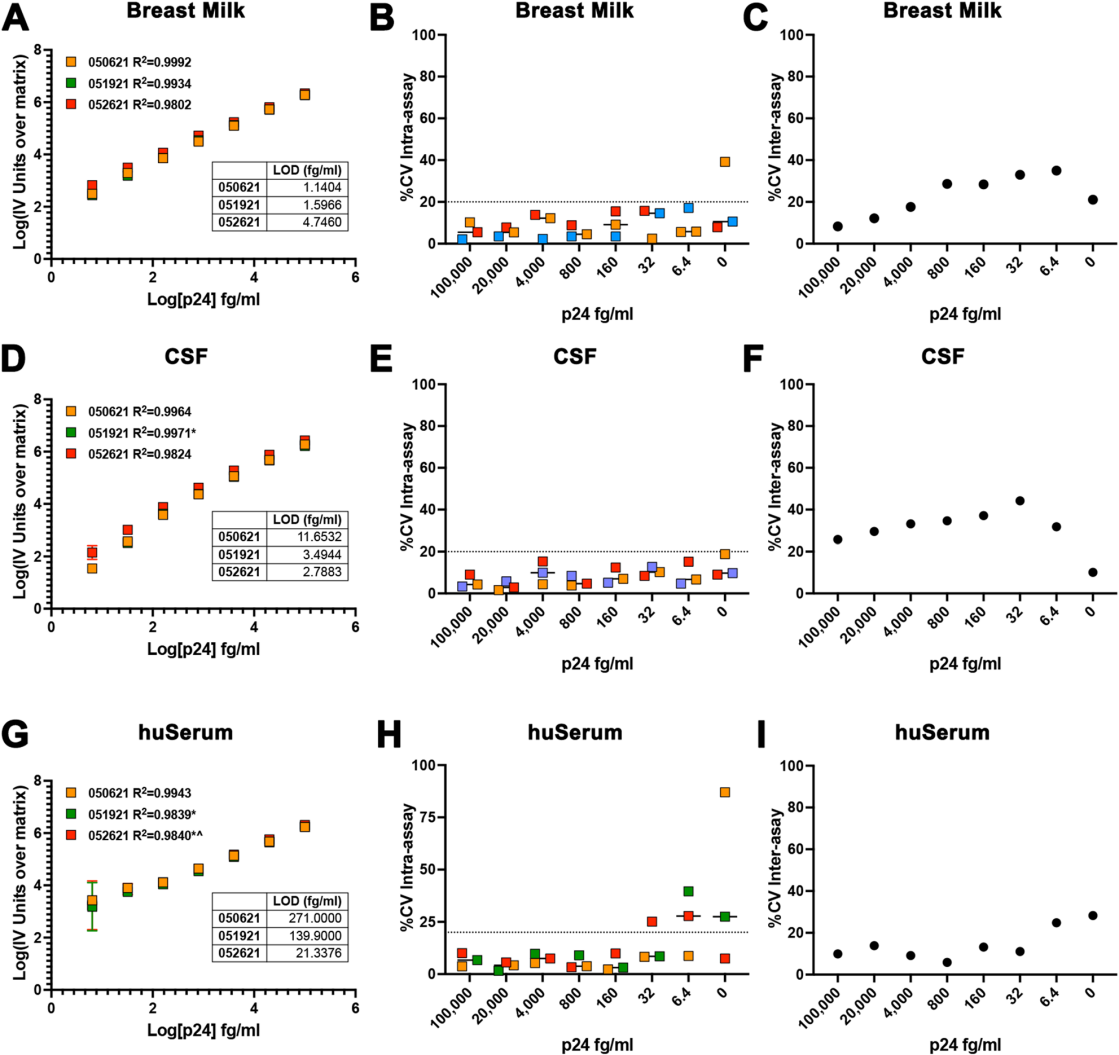
Evaluation of the homebrew Simoa planar p24 ELISA immunoassay in diverse biological fluids. Standard curves of 3 independent experiments using a range of concentrations from 100 pg/ml to 6.4 fg/ml of p24 prepared in breast milk (**A**), cerebrospinal fluid (CSF) (**D**) or human serum (huSerum) (**G**). Each standard was done in technical triplicates. Data has been transformed by subtracting the IV units from the matrix (0) and calculating the log10 from both the IV units and the p24 concentration. Low limit of detection (LOD) for each experiment is provided as table. % of the coefficient of variation (%CV) intra-assay for each concentration for the 3 independent experiments in breast milk (**B**), CSF (**E**) or huSerum (**H**). % of the coefficient of variation (%CV) inter-assay for each concentration for breast milk (**C**), CSF (**F**) or huSerum (**I**). *Optimized curves had to be generated removing the 6.4 fg/ml standard to produce an R^2^. ^Optimized curves had to be generated removing the 32 fg/ml standard to produce an R^2^.

First, we evaluated the compatibility of the assay with biological fluids in which HIV-1 can be found including breast milk, CSF, and huSerum. We diluted the HIV p24 antigen directly in commercially available fluids from HIV negative donors. Both breast milk and CSF performed with high sensitivity and low variability similar to the sample diluent (Fig. 2A-F). Breast milk had a LOD of 2.49 ± 1.96 fg/ml and CSF of 5.98 ± 4.92 fg/ml. On the other hand, huSerum reduced the sensitivity of the assay with an LOD of 144.1 ± 124.9 fg/ml (Fig. 2G-I).

**Figure 3:**
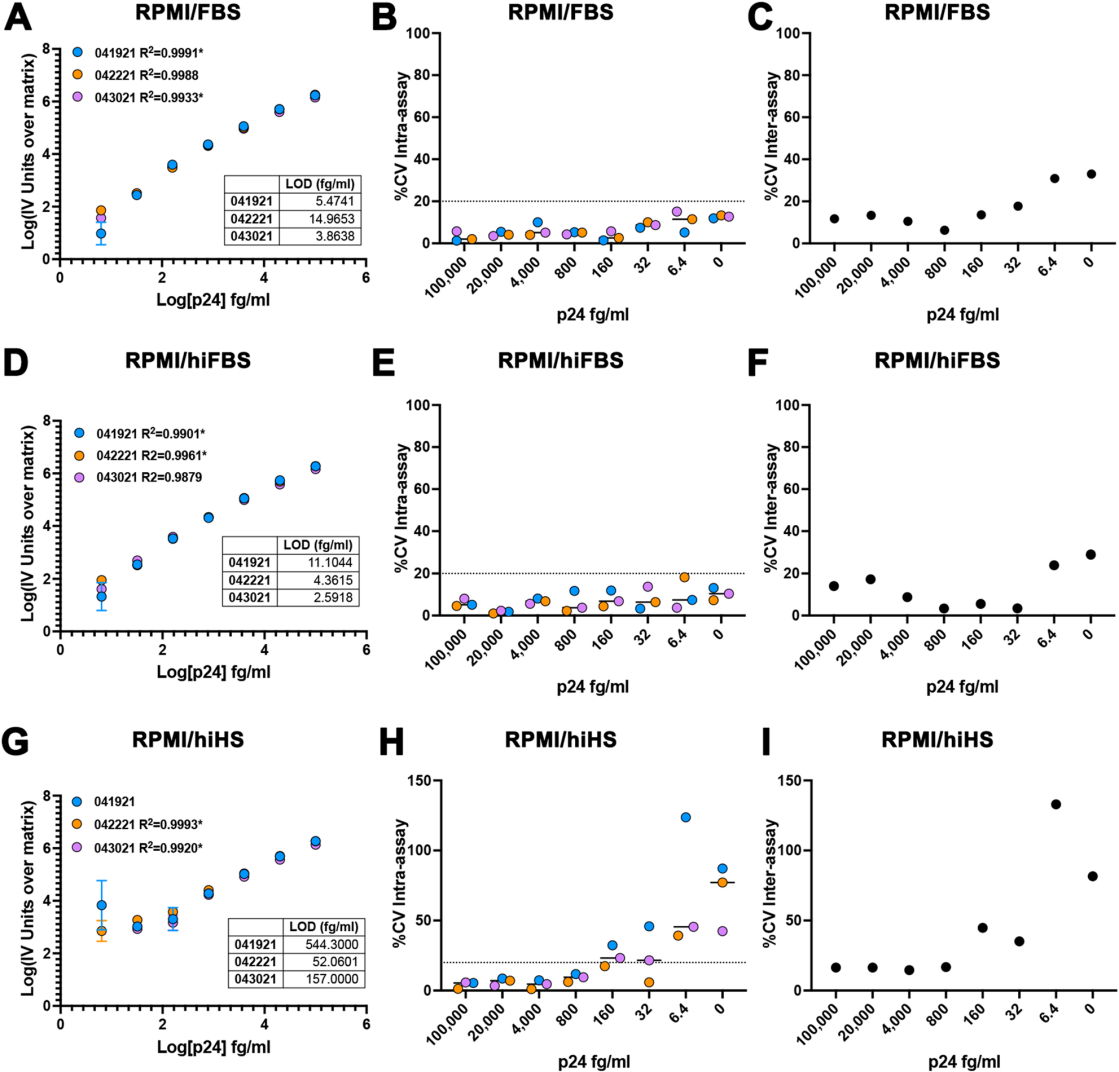
Evaluation of the homebrew Simoa planar p24 ELISA immunoassay in tissue culture media. Standard curves of 3 independent experiments using a range of concentrations from 100 pg/ml to 6.4 fg/ml of p24 prepared in RPMI + 10% FBS (RPMI/FBS) (**A**), RPMI + 10% heat inactivated FBS (RPMI/hiFBS) (**D**) or RPMI + 10% Heat inactivated HS (RPMI/hiHS) (**G**). Each standard was done in technical triplicates. Data has been transformed by subtracting the IV units from the matrix (0) and calculating the log10 from both the IV units and the p24 concentration. Low limit of detection (LOD) for each experiment is provided as table. % of the coefficient of variation (%CV) intra-assay for each concentration for the 3 independent experiments in RPMI/FBS (**B**), RPMI/hiFBS (**E**) or RPMI/hiHS (**H**). % of the coefficient of variation (%CV) inter-assay for each concentration for RPMI/FBS (**C**), RPMI/hiFBS (**F**) or RPMI/hiHS (**I**). *Optimized curves had to be generated removing the 6.4 fg/ml standard to produce an R^2^.

Next, we evaluated the sensitivity and reproducibility of the assay in tissue culture media. We, and others, have previously evaluated the digital ELISA to measure p24 release upon stimulation of cells isolated from aviremic PLWH with different LRAs^20,22,25^. As such, being able to measure p24 using a lower volume with a similar sensitivity can potentially allow the performance of viral release kinetics upon stimulation^20^. Furthermore, preparing the standard in the same media as the samples could reduce the generation of false positives due to interferences of the media with the assay. We tested commonly used media using RPMI plus either 10% fetal bovine serum (FBS), 10% heat inactivated FBS (hiFBS) or 10% heat inactivated pooled A/B human serum (hiHS). We diluted the HIV-1 p24 antigen directly in the corresponding media. The assay was highly sensitive and reproducible in media containing either FBS or hiFBS (Fig. 3A-F). The assay seemed to perform slightly better in hiFBS than in FBS with LOD of 6.02 ± 4.49 fg/ml versus 8.10 ± 5.99 respectively, suggesting that the presence of complement could interfere with the sensitivity of the assay. The media containing hiHS did interfere with the sensitivity and reproducibility of the assay at concentrations below 160 fg/ml and increased the LOD to 251.1 + 259.3 fg/ml (Fig. 3G-I).

**Figure 4:**
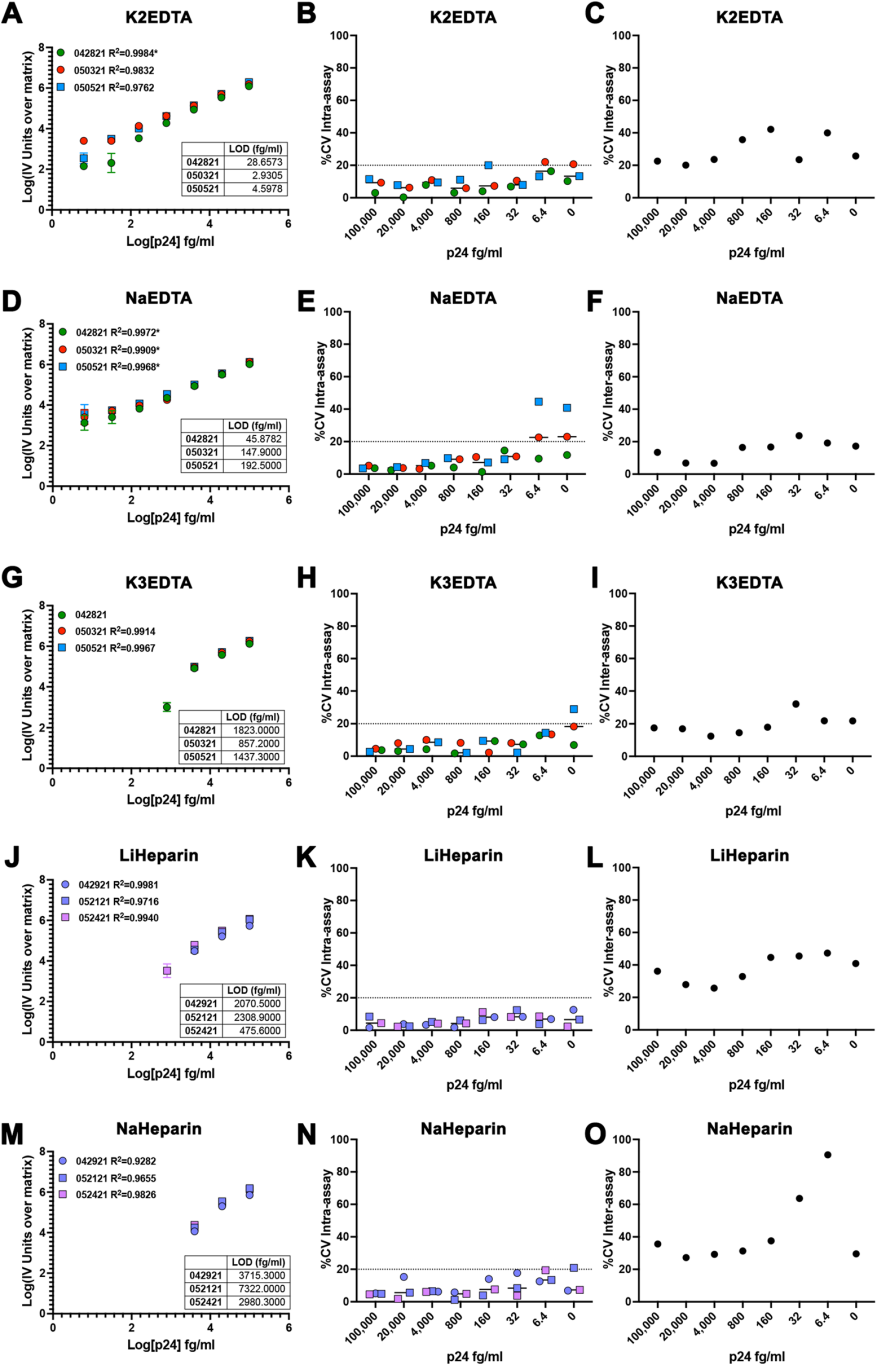
Evaluation of the homebrew Simoa planar p24 ELISA immunoassay in plasma with different anticoagulants. Standard curves of 3 independent experiments using a range of concentrations from 100 pg/ml to 6.4 fg/ml of p24 prepared in K2EDTA plasma (**A**), NaEDTA plasma (**D**), K3EDTA plasma (**G**), LiHeparin plasma (**J**) or NaHeparin plasma (**M**). Each standard was done in technical triplicates. Data has been transformed by subtracting the IV units from the matrix (0) and calculating the log10 from both the IV units and the p24 concentration. Low limit of detection (LOD) for each experiment is provided as table. % of the coefficient of variation (%CV) intra-assay for each concentration for the 3 independent experiments in K2EDTA plasma (**B**), NaEDTA plasma (**E**), K3EDTA plasma (**H**), LiHeparin plasma (**K**) or NaHeparin plasma (**N**). % of the coefficient of variation (%CV) inter-assay for each concentration for K2EDTA plasma (**C**), NaEDTA plasma (**F**), K3EDTA plasma (**I**), LiHeparin plasma (**L**) or NaHeparin plasma (**O**). *Optimized curves had to be generated removing the 6.4 fg/ml standard to produce an R^2^.

Finally, we evaluated whether the assay will be compatible with plasma samples. Recently, the digital ELISA has been used to evaluate p24 in plasma samples from HIV-infected individuals^18^. To that end, we diluted the HIV p24 antigen directly in plasma generated with different anticoagulants including K2EDTA, NaEDTA, K3EDTA, LiHeparin, NaHeparin, and NaCitrate (Fig. 4). From all the plasmas tested, K2EDTA plasma had the highest sensitivity and reproducibility with an LOD of 12.06 ± 14.40 fg/ml (Fig. 4A-C), followed by NaEDTA plasma with an LOD of 128.8 ± 75.16 fg/ml (Fig. 4D-F). K3EDTA, LiHeparin, and NaHeparin plasma had LOD in the low pg/ml (Fig. 4G-O). NaCitrate plasma was incompatible with the assay at the concentrations tested (Extended Data Fig. 2 and Table I). These results suggest than K2EDTA will be the most suitable anticoagulant to use when evaluating p24 directly in plasma samples isolated from PLWH. In conclusion, we demonstrated that the homebrew Simoa planar p24 ELISA can be performed in multiple different matrixes with LODs in the low fg/ml.

### High background due to the matrix reduces the sensitivity of the homebrew Simoa planar p24 ELISA

**Figure 5:**
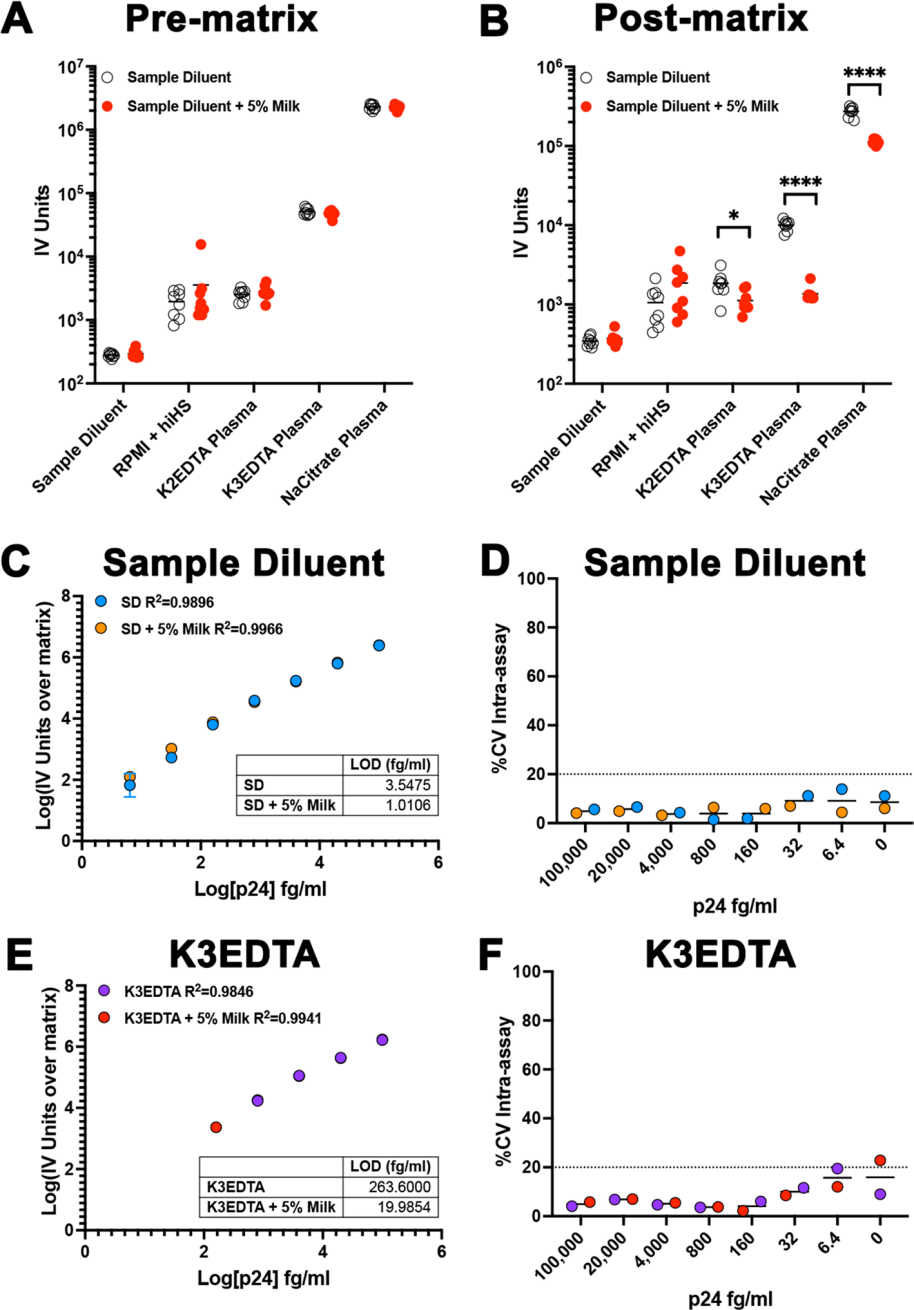
Addition of an extra blocking step after matrix incubation improves the sensitivity of the assay. An additional blocking step was introduced before (**A**) or after (**B**) incubation with the indicated matrixes. Unpaired t test was used to calculate *p* values (*< 0.05; ****< 0.0001). Standard curve using a range of concentrations from 100 pg/ml to 6.4 fg/ml of p24 prepared in sample diluent (**C**) or K3EDTA plasma (**E**) using a blocking step with either sample diluent or sample diluent supplemented with 5% milk. Each standard was done in technical triplicates. Data has been transformed by subtracting the IV units from the matrix (0) and calculating the log10 from both the IV units and the p24 concentration. Low limit of detection (LOD) for each experiment is provided as table. % of the coefficient of variation (%CV) intra-assay for each concentration in sample diluent (**D**) or K3EDTA plasma (**F**)**.**

Next, we wanted to further understand the factors that contribute to sensitivity loss of the homebrew planar p24 ELISA with different matrixes. Both the LOD and the Limit of quantification (LOQ) differ among the diverse matrixes evaluated (Extended Data Fig. 2A,B). We also observed the light intensity (IV) units measured with each of the matrix blank controls differs among matrixes (Extended Data Fig. 2C). In fact, the background IV units were highly correlated with the LOD (Extended Data Fig. 2D) and LOQ (Extended Data Fig. 2E) for each matrix. Furthermore, both the LOD and LOQ were highly correlated (Extended Data Fig. 2F). This result suggests that the background IV units associated with the matrix could be a strong driver in the sensitivity of the planar array assay. One of the potential explanations of the high background could be due to non-specific binding of matrix proteins, the detector antibody, or the streptavidin HRP. To address whether the background associated with the matrixes could be reduced, and the sensitivity of the assay increased, we introduced a blocking step using sample diluent supplemented with 5% milk either prior to or after incubation of the matrix. We decided to use milk as our previous results indicated that breast milk does not interfere with the assay (Fig. 2 and Extended Data Fig. 2). Albeit we did not appreciate a background reduction when the step was added prior to the matrix (Fig. 5A), we did observe a significant background reduction with different plasma matrixes when the blocking step was performed after the matrix incubation (Fig. 5B). We next addressed whether this reduction in matrix background could improve the sensitivity of the assay. We prepared the p24 standard in either sample diluent or K3EDTA plasma and performed the assay introducing the blocking step after incubation with the matrix with either sample diluent alone or with sample diluent supplemented with 5% milk. The additional blocking step did not interfere with the assay using sample diluent as matrix for the standard (Fig. 5C-D). Interestingly, adding the extra blocking step with milk improved the LOD 3.5-fold. The improvement in the sensitivity of the assay was more evident for K3EDTA plasma, reducing the LOD 13-fold to the low fg/ml, without interference in the reproducibility of the assay (Fig. 5E-F). In conclusion, the addition of a blocking step after matrix incubation can improve the sensitivity of the homebrew planar p24 ELISA.

### Validation of the homebrew Simoa planar p24 ELISA

**Figure 6:**
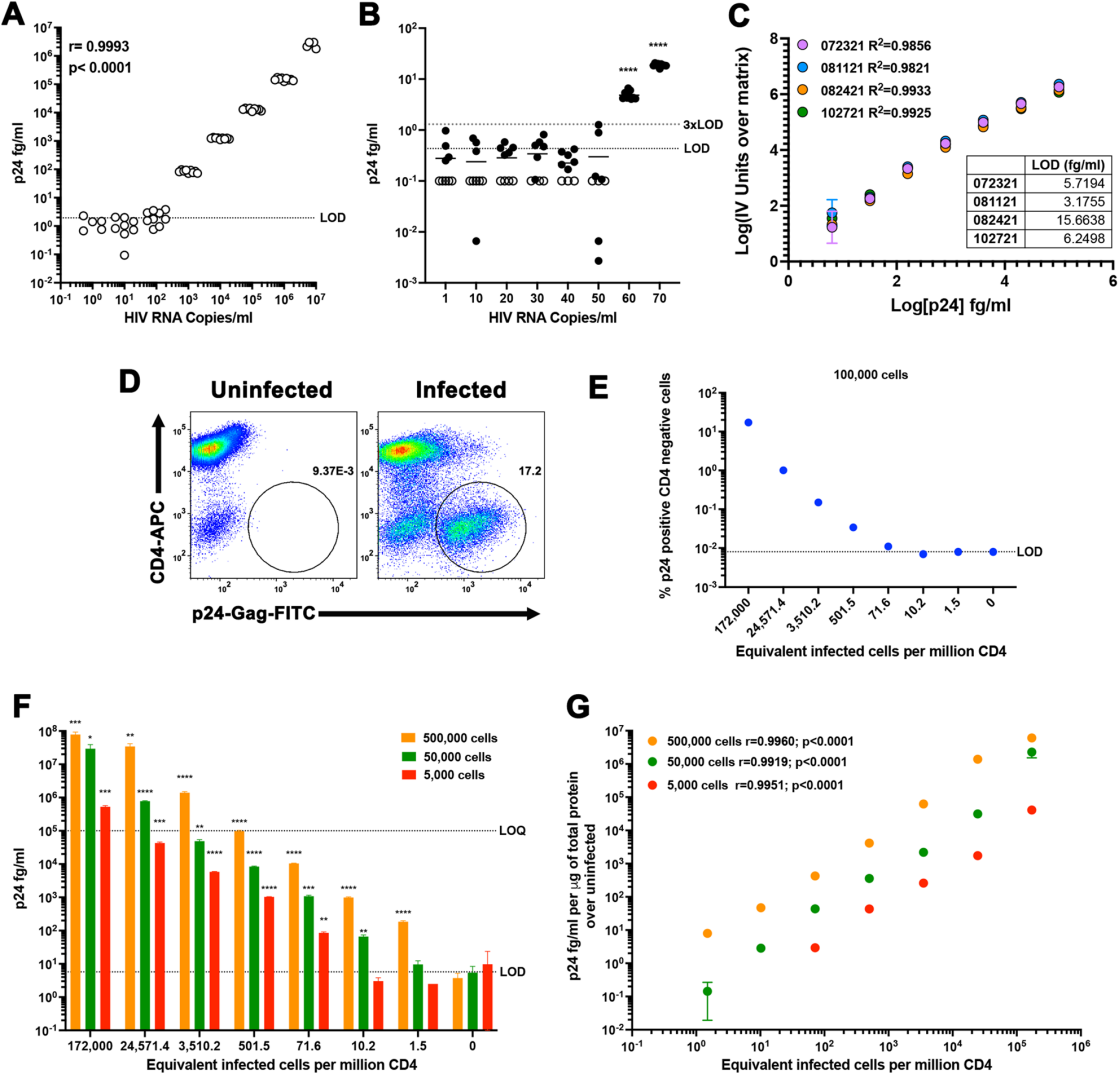
Validation of the homebrew Simoa planar p24 ELISA. (**A**) Nine replicates of tenfold serial dilutions of a viral stock of JR-CSF were quantified using a standard curve with a range of concentrations from 100 pg/ml to 6.4 fg/ml of p24 prepared in sample diluent. Correlation was calculated using Pearson correlation. (**B**) Nine replicates of the indicated HIV-1 RNA copies were quantified using a standard curve with a range of concentrations from 100 pg/ml to 6.4 fg/ml of p24 prepared in sample diluent. Open symbols were identified to be below the limit of detection of the assay. One sample t test was used to calculate *p* values over the LOD (****< 0.0001). (**C**) Standard curves of 4 independent experiments using a range of concentrations from 100 pg/ml to 6.4 fg/ml of p24 prepared in NETN. Each standard was done in technical triplicates. Data has been transformed by subtracting the IV units from the matrix (0) and calculating the log10 from both the IV units and the p24 concentration. Low limit of detection (LOD) for each experiment is provided as table. (**D**) Primary CD4 T cells were infected with JR-CSF and levels of infection analyzed by flow cytometry. (**E**) Seven-fold dilution of infected cells in uninfected cells were analyzed by flow cytometry. The number of equivalent infected cells per million CD4T cells was calculated as indicated in the online Methods. (**F**) Lysates of each dilution corresponding to 500,000, 50,000 or 5,000 cells were quantified using a standard curve using a range of concentrations from 100 pg/ml to 6.4 fg/ml prepared in NETN buffer. Unpaired t test was used to calculate *p* values relative to uninfected (*< 0.05, **< 0.01; ***< 0.001, ****< 0.0001). (**G**) Correlation between the levels of p24 per μg of total protein and the equivalent infected cells per million CD4 was calculated using Pearson correlation.

Next, we wanted to validate whether the assay can detect both viral particles as well as HIV-infected primary CD4T cells. First, viral stocks of the JR-CSF viral strain of HIV-1 were quantified using qPCR. A serial dilution of the viral stock was made from 10^7^ to 1 HIV copies/ml in assay diluent and the levels of p24 were quantified using the optimized homebrew Simoa planar p24 ELISA. There was a strong linear correlation between the levels of HIV-1 p24 and the levels of HIV-1 RNA with detection above the LOD between 10 and 100 HIV copes per ml (Fig. 6A). To further investigate the limit of detection of HIV-1 RNA copies, we evaluated a range of concentrations spanning 1 to 70 copies/ml. We could clearly detect HIV-1 p24 above the LOD and 3xLOD with as low as 60 HIV RNA copies/ml (Fig. 6B). Since each virion contains 2 HIV-1 RNA copies, our results suggest we could detect as low as 30 virions/ml or 1.5 viral particles in 50 μl of assay diluent, similar to our previous estimation of 1.18 ± 0.98 using purified protein (Fig. 1B).

Next, we wanted to explore whether this assay could be compatible using cell extracts. This will also allow to measure translational competent virus directly in cells. We first evaluated whether the assay was compatible with a cell lysis buffer containing Nonidet P-40 (NP40), EDTA, Tris, and NaCl (NETN). We performed a series of 4 biological replicates using a range of concentrations spanning from 100 pg/ml to 6.4 fg/ml in triplicates and a blank control in NETN. NETN buffer had a LOD of 7.7 + /5.47 fg/ml (Fig. 6C and Extended Table I). Next, we infected primary CD4T cells with the replication competent viral stain of HIV-1 JR-CSF and infection was evaluated using flow cytometry (Fig. 6D). A seven-fold serial dilution of infected cells was done in uninfected primary CD4T cells. A subset of cells in each dilution were stained, and 100,000 alive cells were collected and the percentage of infection was evaluated using flow cytometry. The rest of the cells were lysed and the levels of p24 were evaluated using the homebrew planar p24 ELISA. We calculated the equivalent infected cells per million CD4T cells using the percentage of infection in the infected culture and plotted that against each of the analyses (Fig. 6D). Flow cytometry allows the detection above the LOD between 501 and 71 infected cells per million (Fig. 6E). On the other hand, cell lysates equivalent to 500,000 cells in 50 μl allows the detection of at least 1.5 infected cell equivalent per million CD4T cell (Fig. 6F). Importantly, cell extracts of uninfected cells did not show p24 levels above the LOD, indicating low cross-reactivity of the assay with cellular proteins (Fig. 6F). We then performed serial dilutions of the cell extracts in NETN buffer. We could easily detect as low as 10 and 71 infected cells per million when using extracts of only 50,000 or 5000 cells respectively (Fig. 6F). Finally, we normalized the levels of p24 to the total amount of protein calculated using a bicinchoninic acid (BCA) protein assay. We observed a strong correlation between the levels of p24 protein and the levels of infected cells at the three dilutions with detection of 1.5 infected cell equivalents above uninfected cells using cell extracts of either 500,000 or 50,000 cells (Fig. 6G).

**Figure 7:**
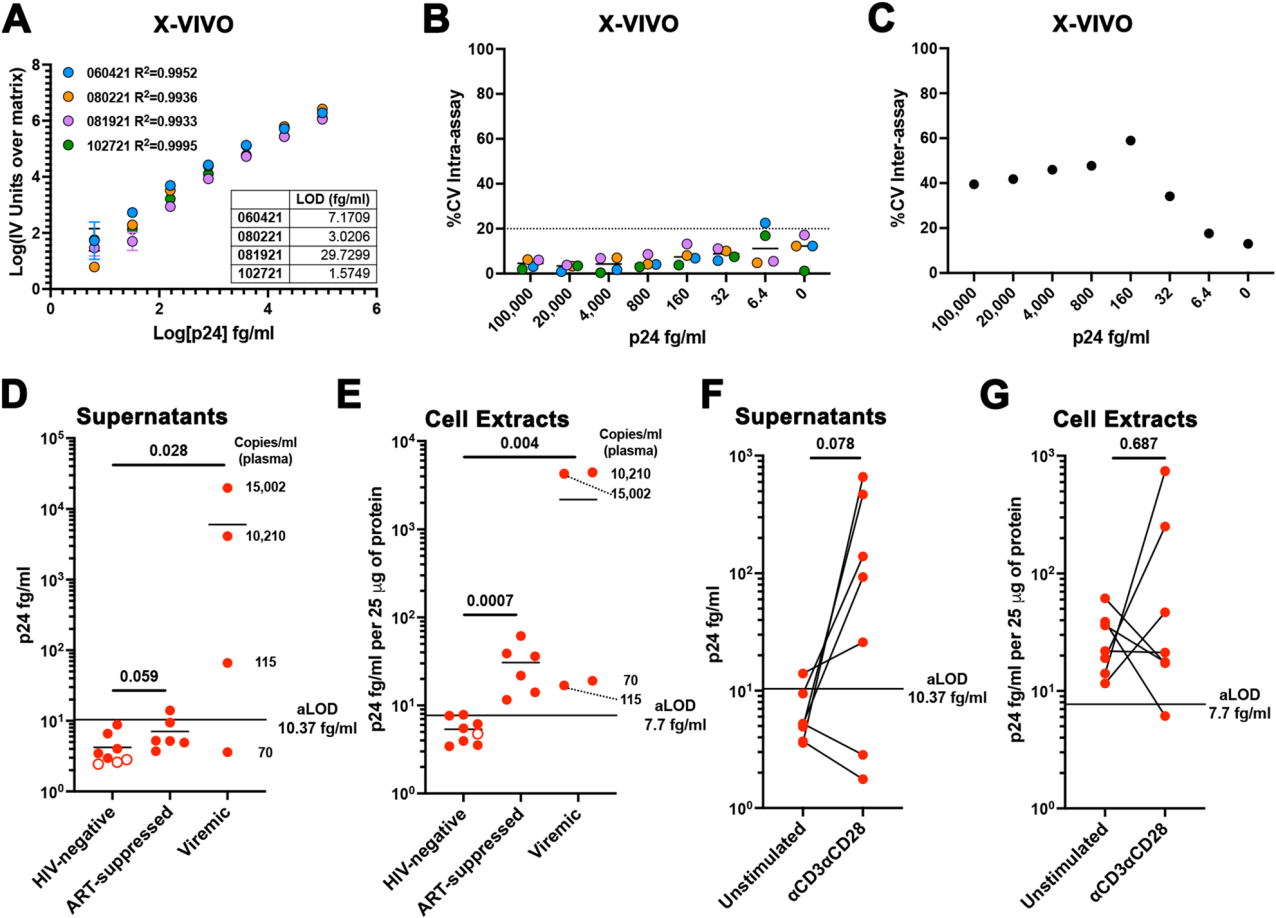
Evaluation of the homebrew Simoa planar p24 ELISA in cells isolated from PLWH. (**A**) Standard curves of 4 independent experiments using a range of concentrations from 100 pg/ml to 6.4 fg/ml of p24 prepared in X-VIVO media. Each standard was done in technical triplicates. Data has been transformed by subtracting the IV units from the matrix (0) and calculating the log10 from both the IV units and the p24 concentration. Low limit of detection (LOD) for each experiment is provided as table. (**B**) % of the coefficient of variation (%CV) intra-assay for each concentration for the 4 independent experiments. (**C**) % of the coefficient of variation (%CV) inter-assay for each concentration. Levels of p24 in the supernatant (**D**) or cell lysates (**E**) of HIV-negative, ART-suppressed and viremic PLWH. Copies per ml in plasma of the viremic participants is indicated. Levels of p24 in the supernatant (**D**) or cell lysates (**E**) ART-suppressed PLWH upon reactivation with αCD3αCD28. Wilcoxon matched-pairs signed rank test was used to calculated *p* values.

Finally, we evaluated whether the assay could detect HIV-1 p24 in cells isolated from PLWH in vitro. To that end, we first evaluated whether the assay was compatible with a serum free media as serum could influence the sensitivity of the assay (Fig. 3). We used the X-VIVO 15 Hematopoietic Serum-Free Culture (X-VIVO) media, which supports the proliferation of lymphocytes under serum-free conditions. We performed a series of 4 biological replicates using a range of concentrations spanning from 100 pg/ml to 6.4 fg/ml in triplicates and a blank control in X-VIVO media. X-VIVO media performed with high sensitivity and low variability similar to the sample diluent, with a LOD of 10.37 ± 13.12 (Fig. 7A-C and Extended Table I). Next, 10^7^ PMBCs isolated from eight HIV-negative donors, six ART-suppressed, or four viremic PLWH were cultured for 4 days in 3 ml of X-VIVO media. After four days, HIV-1 p24 was quantified in both the supernatants and cell lysates prepared as indicated in the Methods section. All the supernatants collected from HIV-negative and ART-suppressed samples were under the limit of detection of the assay (Fig. 7D and Extended Data Fig. 3A). We could easily detect HIV-1 p24 in the supernatants from cells isolated from viremic PLWH. The level of viral release was associated with the levels of viremia in plasma, with higher viral release found in those participants with higher levels of plasma viremia. On the other hand, HIV-1 p24 could be detected above the LOD in all the cell lysates from PLWH regardless of whether they were ART-suppressed or viremic, but not for any of the HIV-negative donor samples (Fig. 7E and Extended Data Fig. 3B). These results indicate that some levels of HIV Gag expression can still be detected in cells even in the absence of detectable viremia in plasma or viral release in culture. Lastly, we wanted to evaluate whether this assay could be used to detect reactivation of the latent reservoir. To that end, cells from the 6 ART-suppressed PLWH and the viremic patient with the lowest levels of viremia in plasma were stimulated with αCD3/αCD28. Supernatants and cell lysates were collected 4 days later. We could easily detect viral reactivation in the supernatants of 5 out 7 participants (Fig. 7F and Extended Data Fig. 3C). Interestingly, we did not observe major differences in HIV p24 in the cell extracts. In 3 participants the levels went up, in 3 went down and one did not change. (Fig. 7G and Extended Data Fig. 3D).

In conclusion, we validated the homebrew planar p24 ELISA and demonstrated that the assay could detect HIV-1 p24 both in viral particles and in cell lysates with a limit of detection similar to nucleic acid assays and can detect HIV-1 p24 using cells isolated from PLWH enhancing the versatility of the assay.

## Discussion

The development of ultrasensitive assays to measure HIV-1 protein in diverse biological fluids can add to the repertoire of assays used to evaluate the efficacy of different clinical interventions towards an HIV-1 cure. As of today, only one assay is available that detects HIV-1 Gag protein to that of the levels of RNA by using a dELISA^16,17^. This assay uses beads to measure protein concentration using single-molecule analysis allowing limits of detection in the fg/ml. In this study, we have evaluated a planar array immunoassay. This assay uses an anchor antibody microprinted in 96-well plates and offers high-sensitivity, flexibility and affordability. This assay’s full protocol can be performed in approximately 5 hour (Extended Data Fig. 4). Furthermore, a cost analysis of the assay shows the cost per sample ranging only from $3 to $6 per replicate in function of the matrix used to prepare the standards (Extended Table II). Finally, our results demonstrate that the homebrew planar p24 ELISA allows the detection of HIV-1 p24 to levels comparable to that of the dELISA and with LOD similar to standard nucleic acid assays.

This assay has several advantages over the previous one. First, this new assay allows the detection of HIV p24 directly in different biological matrixes without appreciable loss of sensitivity or reproducibility, including breast milk, CSF and cell lysates. Second, it uses less volume and it does not require further manipulation of the sample. Third, it allows higher accuracy of quantification by using the same matrix to prepare the standard as the sample. Our study also revealed that the anticoagulant used to obtain plasma can have a major influence in the sensitivity of the assay. Based on our studies, K2EDTA plasma will be the recommended anticoagulant if no further manipulation of the sample such as dilution or precipitation can be performed^19^. As such, this assay could be used to evaluate cure strategies currently in clinical trials such as “shock-and-kill” to measure HIV-1 p24 in diverse biological fluids and/or cell lysates after LRA administration using limited amount of sample.

This assay can also be performed directly using tissue culture media. For example, it could be used to evaluate the LRA activity of compounds in preclinical development or to evaluate the size of the replication competent reservoir calculating infectious units per million (IUPM), as recently done with the dELISA (DEVO assay)^20–22,25^; or to evaluate increase in HIV protein translation ex vivo using cell lysates. Our results suggest that serum in the media can reduce the sensitivity of the assay by increasing the background. We evaluated a serum-free media (X-VIVO) and demonstrated that the assay is compatible with this media. Then, we performed a proof-of-principle study using a limited amount of sample, 10^7^ PBMCs, isolated from PLWH. We demonstrate that viral release could be detected after a 4-day culture in viremic participants but not ART-suppressed, or upon antigen stimulation. HIV-1 p24 could be readily detected in cell lysates from 10^6^ PBMCs in all the participants but not in the HIV-negative donors regardless of the detection of viral release, or whether the participant was ART-suppressed or viremic. Interestingly, no changes in HIV-1 p24 in the cell lysates were detected upon antigen stimulation. One potential reason that can account for this result is that upon 4 days of stimulation we could only detect new virion release but not increase in viral replication. Further studies are warranted to address this dichotomy.

Our study has some limitations. First, the sensitivity or reproducibility of the assay in other tissue culture medias (i.e. DMEM, MEM, Opti-MEM, IMDM), other serum (i.e. FCS) or serum concentrations, other potential biological fluids (i.e. seminal or vaginal fluid) or other cell lysate buffers were not tested. Further evaluation will be required to assess the compatibility of the assay in these other matrixes if necessary. Second, other potential anti-HIV-1 Gag antibodies were not tested. The homebrew assay allows the optimization for any pair of capture/detector antibody and it may be possible to use a different pair to obtain similar sensitivity and reproducibility. Third, the gag protein used for the standard is a heat-inactivated viral antigen from HIV-1IIIB from a commercially available and validated ELISA. Other commercially available gag proteins, other clades, or HIV-2 gag (p26) were not tested and further development will be required. The capture antibody used recognizes HIV-2 and other detector antibodies could be used to specifically detect HIV-2. Finally, the assay loses sensitivity with those matrixes that have a high background probably due to non-specific binding of either the detector antibody or the streptavidin HRP to the matrix. Interestingly, adding an extra blocking step can improve the sensitivity of the assay in some of those instances. However, only 5% milk was tested and other blocking reagents or concentration of the blocking reagent may also improve the sensitivity of the assay. Further optimization to mitigate the matrix effect will be required on those instances in which the matrix may interfere with the assay and the additional blocking step does not improve it. In spite of these caveats, here we demonstrate that the homebrew planar p24 ELISA could be a potential new tool and assay that can be used to evaluate HIV-1 cure approaches and persistence in different biological fluids and cell lysates, in particular when sample availability is limited.

## Methods

### Reagents

The following matrixes were obtained from Innovative Research: Pooled Human Plasma (blood derived) with the anticoagulants K2 EDTA (cat# IPLAWBK2E), K3 EDTA (cat# IPLAWBK3E), Na EDTA (cat# IPLAWBNAE), Li Heparin (cat# IPLAWBLIH5), Na Heparin (cat# IPLAWBNAH), Na Citrate (cat# IPLAWBNAC), Pooled Human Cerebrospinal Fluid (cat# IRHUCSF), Pooled Human Pasteurized Breast Milk (cat# IRHUBMKPST) (pooled with a minimum of three donors), and Pooled Human AB Serum Plasma Derived (cat# ISERAB) (pooled from male donors blood type AB). Anti-HIV type 1 p24 clone 39/5.4A (cat# 0801136) was purchased from Zeptometrix. Monoclonal anti-HIV-1/2 purified antibody (cat# HIV-018-48303) was purchased from Capricorn Products LLC. The following reagents were obtained from Thermo Fisher Scientific: Sulfo-SMCC (cat# A39268), NHS-PEG_4_-Biotin (cat# A39259) and Ultrapure 1 M Tris–HCl (cat# 15-567-027). Simoa Planar Array Homebrew Tag 1, Sample Diluent A, Streptavidin-HRP, Stable Peroxide, SuperSignal Luminol, Homebrew plate, and 25X wash buffer were all obtained from Quanterix. The following reagent was obtained through the NIH HIV Reagent Program, Division of AIDS, NIAID, NIH: Human Immunodeficiency Virus 1 (HIV-1), Strain JR-CSF Infectious Molecular Clone (pYK-JRCSF), ARP-2708, contributed by Dr. Irvin SY Chen and Dr. Yoshio Koyanagi.

### Generation of capture and detector antibodies

The conjugation of the capture and detection antibody with homebrew reagents was performed by following manufacturers protocol. Briefly, capture and detection antibodies were generated through a series of washes in Amicon filters to exchange the antibody buffer to the assay conjugation buffer as indicated in the Simoa Planar Array Homebrew Starter kit. The concentration of each antibody was measured using Nanodrop Spectrophotometer and the concentration adjusted to 1 mg/mL in conjugation buffer. The capture antibody was first tagged using Sulfo-SMCC for 30 min then 3.65 μL of Ultrapure Tris–HCl buffer was added to stop the reaction. Then, the Simoa Planar Array Homebrew Tag 1 was added for 30 min at room temperature. The detection antibody was incubated with NHS-PEG_4_-Biotin for 30 min at room temperature. Both capture and detection antibodies were then purified to remove excess Sulfo-SMCC, Tag 1, and Biotin through a series of washes with an Amicon filter. The concentration of each antibody was measured using Nanodrop Spectrophotometer and the concentration was adjusted to 0.25 mg/mL in the Simoa Planar Array Homebrew Diluent A. The capture and detection antibodies are then stored at 4 °C for at least one month until ready to use.

### Planar array assay procedure

Prior to performing the assay, the 25× wash buffer was diluted to 1× with ddH_2_0. The calibrators were generated by mixing the matrix, 1% Triton X-100, and p24 protein for the top concentration of 100 pg/mL. The calibrators went through serial fivefold dilutions to achieve the following concentrations: 100, 20, 4, 0.8, 0.16, 0.032, 0.0064, and 0 pg/mL totaling eight concentrations for a standard curve. The Microclime lid was filled with 4 mL of ddH_2_O to wet the outer edges of the sponge and used throughout the duration of the assay. The homebrew plate was washed four times using Cappwash manual plate washer and patted dry to remove excess wash buffer from wells. The capture antibody was diluted to 1 μg/mL in sample diluent and 50 μL was added to the wells and incubated for 30 min on a shaker at 515 rpm. After incubation, the plate was washed four times and patted dry to remove excess wash buffer from wells. 50 μL of each calibrator concentration was added to the wells in triplicates and incubated for 2 h on a shaker at 515 rpm. The plate was then washed four times and patted dry to remove excess wash buffer from wells. The detection antibody was diluted to 1 μg/mL in sample diluent and 50 μL was added to the wells and incubated for 30 min on a shaker at 515 rpm. The plate was washed six times and patted dry to remove excess wash buffer from wells. 50 μL of Streptavidin-HRP was added to the wells and incubated for 30 min on a shaker at 515 rpm. The plate was washed six times and patted dry to remove excess wash buffer from wells. The SuperSignal Substrate was prepared by combining 3 mL of Stable Peroxide and 3 mL SuperSignal Luminol Enhancer and 50 μL was added to the wells. The plate was immediately read on the SP-X Imager.

In some instances, an additional blocking step was added. After incubation of the plate with the matrix and washing four times, 50 μL of 5% non-fat dry milk in sample diluent (filtered through a 0.22 μM) was added to the wells and incubated for 30 min on a shaker at 515 rpm. The plate was then washed four times and patted dry to remove excess wash buffer from wells and the assay continues as described above.

### Viral stock generation and quantification by qPCR

JR-CSF virus was generated by calcium phosphate transfection of pYK-JRCSF plasmid into HEK-293T cells. First, HEK-293T cells were cultured in 10 × 10 cm plates to around 70–80% confluency in DMEM medium in 20 ml 10% FBS and L-glutamine. 1-h prior transfection, media was replaced and plates were returned to the incubator. After the one-hour incubation, 25 μg of plasmid per plate was diluted to a final volume of 750 of sterile dH2O, and 250 μl of 1 M CaCl_2_ were added. Immediately, 1 mL of filtered sterilized 2xHBS (140 mM NaCl, 1.5 mM Na_2_HPO_4_·2H_2_O, 50 mM HEPES, pH 7.05) was quickly added to the same tube and vortexed. The final CaPO4-DNA mixture was allowed to sit for 1 min at RT and then added drop by drop throw-out the HEK-293 T cell plate. The plate was gently rocked (up, down, right and left) several times to evenly distribute the mixture. 20 μL of 100 mM Chloroquine was added to the plate and gently rocked again. The HEK-293T cell plates were incubated overnight at 37 °C and the media was replaced the following day. Supernatants of the plates were collected 36–48 h later and spun down at 2000 rpm for 10 min. Supernatants were then filtered through 0.4 μm filter, aliquoted in cryovials, and stored at − 80 °C for future use.

RNA from viral stock was isolated using the QIAamp Viral RNA Mini Kit following the manufacturer’s protocol (Qiagen, Cat.# 52904). Real-time RT-PCR was performed using the AgPath-ID One Step RT-PCR Kit (Cat# 4387424, Thermo-Fisher Scientific) against a 200-bp amplicon of the HIV pol gene using a forward primer (iSCA-FWD) 5′–TTTGGAAAGGACCAGCAAA-3′, a reverse primer (iSCA-Rev) 5′-CCTGCCATCTGTTTTCCA-3′ (Integrated DNA technologies) and a Taqman TAMRA probe (iSCA probe) 5′-6FAM-AAAGGTGAAGGGGCAGTAGTAATACA-TAMRA-3′ (AB Applied Biosystem)^26^. Real-time PCR was performed using the ViiA 7 Real-Time PCR System (AB Applied Biosystems) as follows: 45 °C for 10 min; 95 °C for 10 min; and 40 cycles of 95 °C for 15 s, 60 °C for 1 min and 72 °C for 30 s. HIV-1 RNA standards were kindly provided by Dr. R. Brad Jones, Cornell University. Briefly, HIV RNA standards were generated by cloning the p31 region of pol from plasmid containing an infectious clone of HIV-1 downstream of the T7 promoter. In vitro RNA synthesis was performed with a 4-h incubation at 37 °C using the MEGAscript T7 Transcription Kit (ThermoFisher)^27^.

### Generation of HIV infected primary CD4T cells and cell lysates

Total CD4 T cells were isolated from HIV-1 negative blood donors using magnetic isolation following the manufacture protocol (EasySep Human CD4 + T Cell Enrichment Kit, STEMCELL Technologies). The isolated total CD4 T cells were activated with 1 αCD3/αCD28 Dynabeads per cell (Cat#11132D, Thermo Fisher Scientific) and plated in 96-well round bottom plates at a density of 0.5 × 10^6^ cells/mL in RPMI supplemented with 10% FBS, penicillin/streptomycin, and L-glutamine (complete media) for 72 h. The cells were then resuspended and transferred to a 15 mL falcon tube before being placed inside the Dynal MPC-L magnetic particle concentrator (Invitrogen) for 1 min to remove the Dynabeads. Cells were transferred to another falcon tube, counted, and spun down at 1500 rpm for 5 min, 4 °C. The cells were then resuspended in complete media at a density of 1 × 10^6^ cells/mL with the addition of 30 IU/mL IL-2. Media plus IL-2 was replaced on days 4 and 5. To generate infected cells, cells were infected on culture day 7 using 129 ng/mL JR-CSF. One-fifth of the culture was kept uninfected in complete media + IL-2. Of the remaining four-fifths, one-fifth was infected with JR-CSF by spinoculation at 2900 rpm for 2 h at 37 °C. After spinoculation, the infected cells were added to the remaining three-fifths culture in complete media plus IL-2. On culture day 10, cells were plated in 96-well round bottom plates in complete media plus IL-2 to facilitate cell-to-cell infection spread for 72 h. On culture day 13, infected and uninfected cells were transferred to 50 mL falcon tubes, counted and resuspended in complete media at a density of 1 × 10^6^ cells/mL. An initial sample of 3.5 × 10^6^ infected cells was serially diluted seven-fold in uninfected cells for 6 times. An eighth sample contained only uninfected cells. All 8 final samples contained 3 × 10^6^ cells in 3 mL complete media. 300,000 cells from each condition were stained for viability, CD4, and p24-Gag so that infection levels could be measured by flow cytometry (see below). The remaining 2.7 × 10^6^ cells per condition were spun down, washed once with sterile PBS, and then resuspended in 270 μL 1 × NETN buffer (0.5% (v/v) Nonidet P-40, 0.5 mM EDTA, 20 mM Tris-Cl pH 8.0, and 100 mM NaCl) supplemented with protease and phosphatase inhibitors (cOmplete, Mini, EDTA-free Protease Inhibitor Cocktail and PhosSTOP™, Sigma-Aldrich).

To generate cell lysates, cells in NETN buffer were incubated on ice for 30 min. To eliminate non-soluble fractions, cell lysates were spun down at 13,000 rpm at 4 °C. The supernatant was then transfer to a clean Eppendorf and store at − 80 °C until analysis. Total protein concentration for each sample was calculated using the BCA assay (Pierce BCA Protein Assay Kit) following the manufacture’s protocol (Cat# 23225, Thermo Fisher Scientific).

### Generation of supernatant and cell lysates from PBMCs isolated from HIV-negative donors and PLWH

Peripheral blood mononuclear cells (PBMC’s) from HIV-negative donors, ART-suppressed and viremic PLWH were cultured at 3.3 million cells per mL in X-VIVO 15 Hematopoietic Serum-Free culture media (Lonza, Cat #04418Q) for four days. After four days, samples were spun down and supernatants were collected and transferred to clean microcentrifuge tube. To prepare cell lysates, 50 μL of NETN buffer per 10^6^ cells was added to the cell pellets and incubated on ice for 30 min. Cell lysates were prepared as described above. In some experiments, PBMCs isolated from PLWH were cultured at 3.3 million cells per mL in X-VIVO 15 as above and activated with CD3 and CD28 mAbs (T cell TransAct, Cat #130-111-160, Miltenyi Biotec, Germany). Cells were cultured for four days and supernatants and cell pellets were collected for HIV p24 measurements.

### Evaluation of HIV infected primary CD4T cells using flow cytometry

For measuring HIV-1 infection by flow cytometry, 3 × 10^5^ cells were first wash with 1 ml PBS, centrifuged 5 min at 1500 rpm, and stained with 0.1 μl of fixable viability dye (eFluor 450, Cat#65-0863-18, ebioscience) in 100 μl of PBS for 10 min at 4 °C. Cells were washed with 1 ml PBS, centrifuged 5 min at 1500 rpm, and stained with 1 μl of anti-CD4-APC (S3.5, APC conjugate, Life Technologies, Cat# MHCD0405) in 100 μl of PBS plus 3% FBS for 30 min at 4 °C. Cells were then washed with 1 ml of PBS plus 3% FBS, centrifuged 5 min at 1500 rpm and fixed and permeabilized with 100 μl of Cytofix/Cytoperm (Cat#554722, BD), vortexed and incubated for 30 min at 4 °C. Cells were then washed with 1 ml of Perm/Wash Buffer (Cat#554723, BD), centrifuged 5 min at 1500 rpm, and stained with 1 μl of anti-HIV Gag-FITC (KC57, FITC conjugate, Beckman Coulter, Cat#C06604665) in 100 μl of Perm/Wash Buffer, vortexed and incubated for 30 min at 4 °C. Cells were then washed with 1 ml of Perm/Wash Buffer (Cat#554723, BD), centrifuged 5 min at 1500 rpm, and resuspended in 2% PFA in PBS after analysis. We defined infected cells by the downregulation of CD4 and expression of p24-Gag, so to determine the positive gate, uninfected cells were stained in parallel. For the 8 experimental samples, 1 × 10^5^ live events were collected. Flow cytometry was performed on a Becton Dickinson LSR Fortessa flow cytometer using FACSDiva acquisition software V8.0.1. FlowJo v10 software was used to analyze the data.

### SP-X imager and analysis

The plate was scanned and analyzed using the Quanterix SP-X Imager. The data file was analyzed using the Quanterix SP-X Analysis Software V2.1.1.7737 where a standard curve using 5 parametric logarithmic (5PL) curve fit, %CV, LOD, LLOQ, and R^2^ were created.

### Study approval

Gulf Coast Regional Blood Center—Volunteers 17 years and older served as blood donors. White blood cell concentrates (buffy coat) prepared from a single unit of whole blood by centrifugation were purchased.

PLWH—The human study was conducted according to the principles expressed in the Declaration of Helsinki. Participants were studied under a MedStar Georgetown University Hospital Institutional Review Board approved HIV clinical research studies CR00000926. PLWH provided written informed consent for the collection of samples and subsequent analysis.

The characteristics of PLWH group (n = 10) used in this study had median age 49, IQR: 40–53 years old and gender distribution was 70% male and 30% female. In this group, six study participants were viral suppressed by combination antiretroviral therapy (cART) and had CD4 counts median 861, IQR: 718–1399 cells/μl and viral load < 40 copies/ml. In addition, four individuals were viremic and had a median CD4 counts of 599, IQR: 454–671 cells/μl, and viral load median 5163, IQR: 81–13,804.

### Statistics

One sample t test or unpaired t test was used to calculate *p* values. Pearson correlation was calculated for correlations. Wilcoxon matched-pairs signed rank test was used for studies using PBMCs. Statistics were calculated using Prism 9 for Mac OS X software (GraphPad).

## Supplementary Information

Supplementary Information.
