# Publisher Correction: An ultrasensitive planar array p24 Gag ELISA to detect HIV-1 in diverse biological matrixes

**DOI:** 10.1038/s41598-021-04119-5

**Published:** 2021-12-21

**Authors:** Callie Levinger, J. Natalie Howard, Jie Cheng, Pingtao Tang, Amit Joshi, Marta Catalfamo, Alberto Bosque

**Affiliations:** 1 Department of Microbiology, Immunology and Tropical Medicine, George Washington University, Washington, USA; 2 Quanterix Corporation, Billerica, MA USA; 3 Department of Microbiology and Immunology, Georgetown University School of Medicine, Georgetown University, Washington, USA

Correction to: *Scientific Reports* 10.1038/s41598-021-03072-7, published online 08 December 2021

The original version of this Article contained an error in the spelling of the author J. Natalie Howard which was incorrectly given as JNatalie Howard.

In addition, in the Abstract,

“In here, we developed an ultrasensitive p24 ELISA that uses the Simoa planar array technology that can detect HIV-1 virions and HIV-1 infected cell with limit of detection similar to nucleic acid assays.”

now reads:

“In here, we developed an ultrasensitive p24 ELISA that uses the Simoa planar array technology that can detect HIV-1 virions and HIV-1 infected cells with limit of detection similar to nucleic acid assays.”

The original article has been corrected.

