# Supplementary material for: An ultrasensitive planar array p24 Gag ELISA to detect HIV-1 in diverse biological matrixes
Source: Sci Rep. 2021 Dec 8;11:23682. doi: 10.1038/s41598-021-03072-7 (PMC8654962; doi:10.1038/s41598-021-03072-7)
Supplement: Supplementary file 1 — Supplementary Information. [file 41598_2021_3072_MOESM1_ESM.docx]

**Extended Data**

**
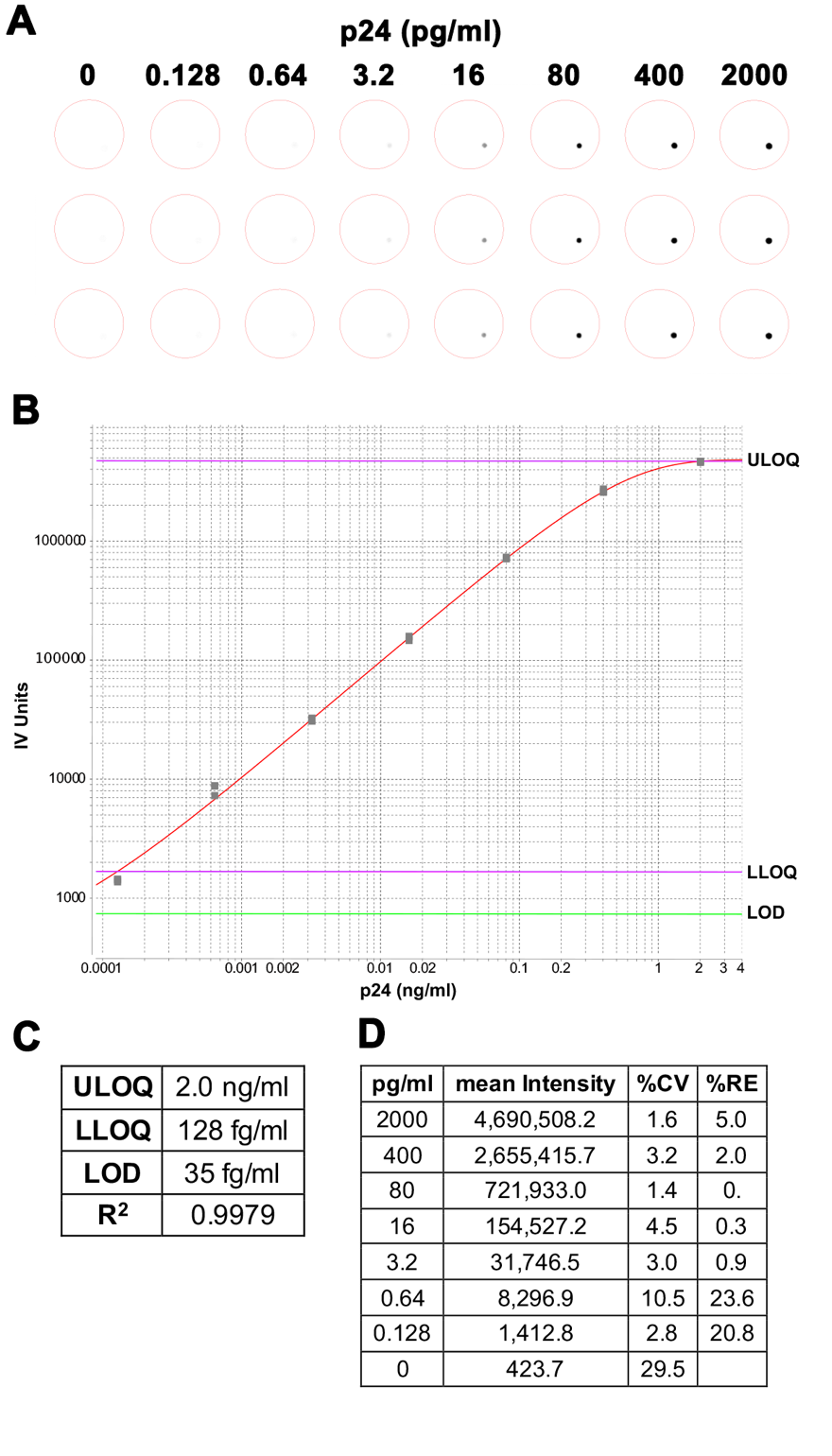
**

**Extended Data Figure 1. The homebrew Simoa planar array p24 ELISA.** **(A)** SP-X image plate of a standard curve of HIV p24 from 2,000 to 0.128 pg/ml. **(B)** Standard curve analyzed using the SP-X analysis software. Upper limit of quantification (ULOQ), low limit of quantification (LLOQ) and low limit of detection (LOD) are indicated with horizontal lines. **(C)** ULOQ, LLOQ, LOD and regression line fit (R^2^) values for the standard curve indicated in **(B)**. **(D)** Mean Intensity, % of the coefficient of variation (%CV) and % of recovery (%RE) for each of the standard curve concentrations shown in **(B)**.

*
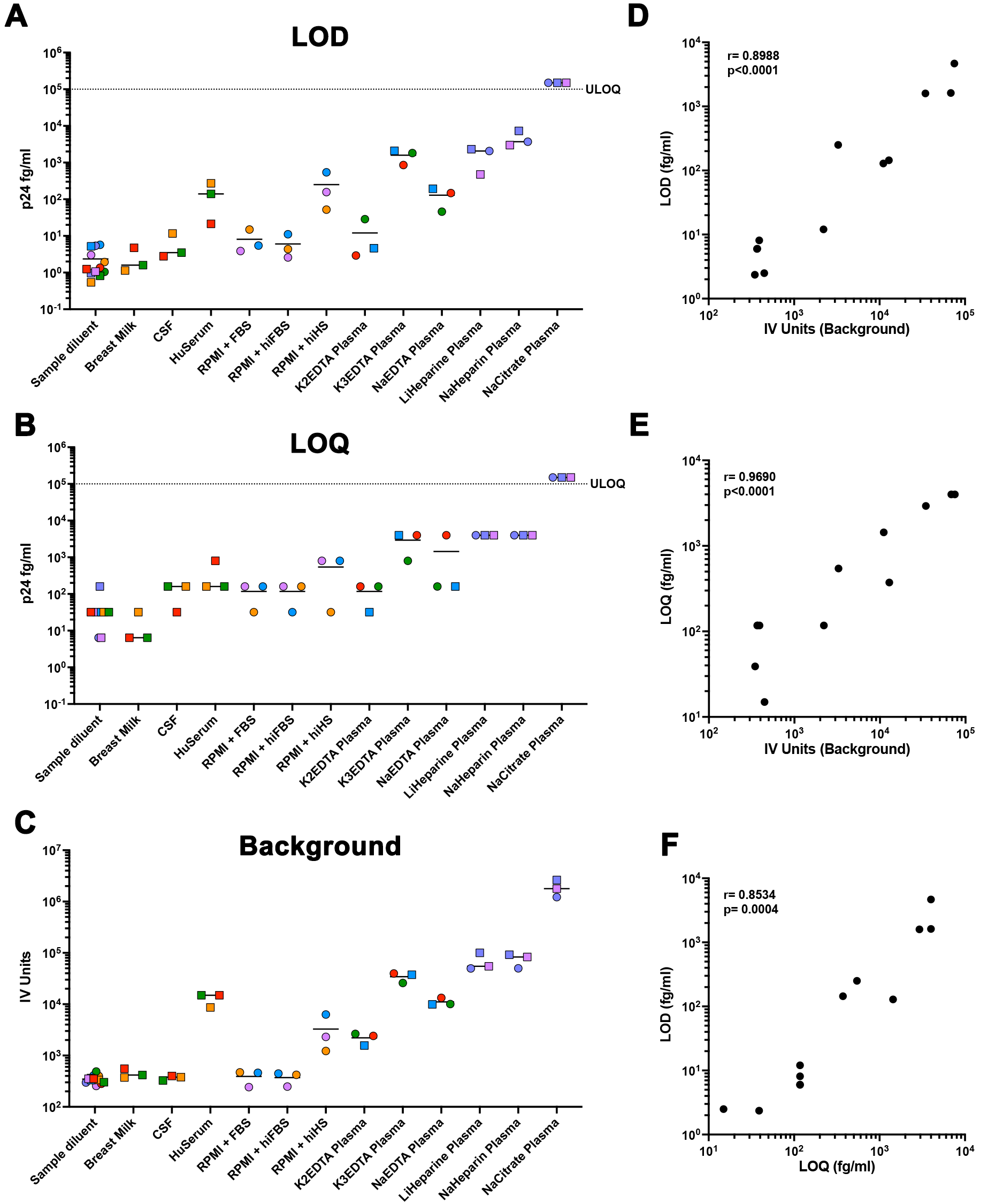
*

**Extended Data Figure 2. Cross-comparison of the homebrew Simoa planar p24 ELISA immunoassay in diverse matrixes**. **(A)** Compilation of the limit of detection (LOD) for each of the matrixes. NaCitrate plasma was above the upper limit of quantification (ULOQ) of the standard. **(B)** Compilation of the limit of quantification (LOQ) for each of the matrixes. NaCitrate plasma was above the upper limit of quantification of the standard. **(C)**. Compilation of the light intensity (IV) units for each of the matrixes background in the absence of p24 protein. **(D)** Correlation of the IV units given by the background in each matrix and the LOD for that matrix, calculated using Pearson correlation. **(E)** Correlation of the IV units given by the background in each matrix and the LOQ for that matrix, calculated using Pearson correlation. **(F)** Correlation of LOD in each matrix and the LOQ for that matrix, calculated using Pearson correlation. NaCitrate plasma was removed of the analysis for **D**, **E** and **F** as the LOD and LOQ was unable to be calculated.


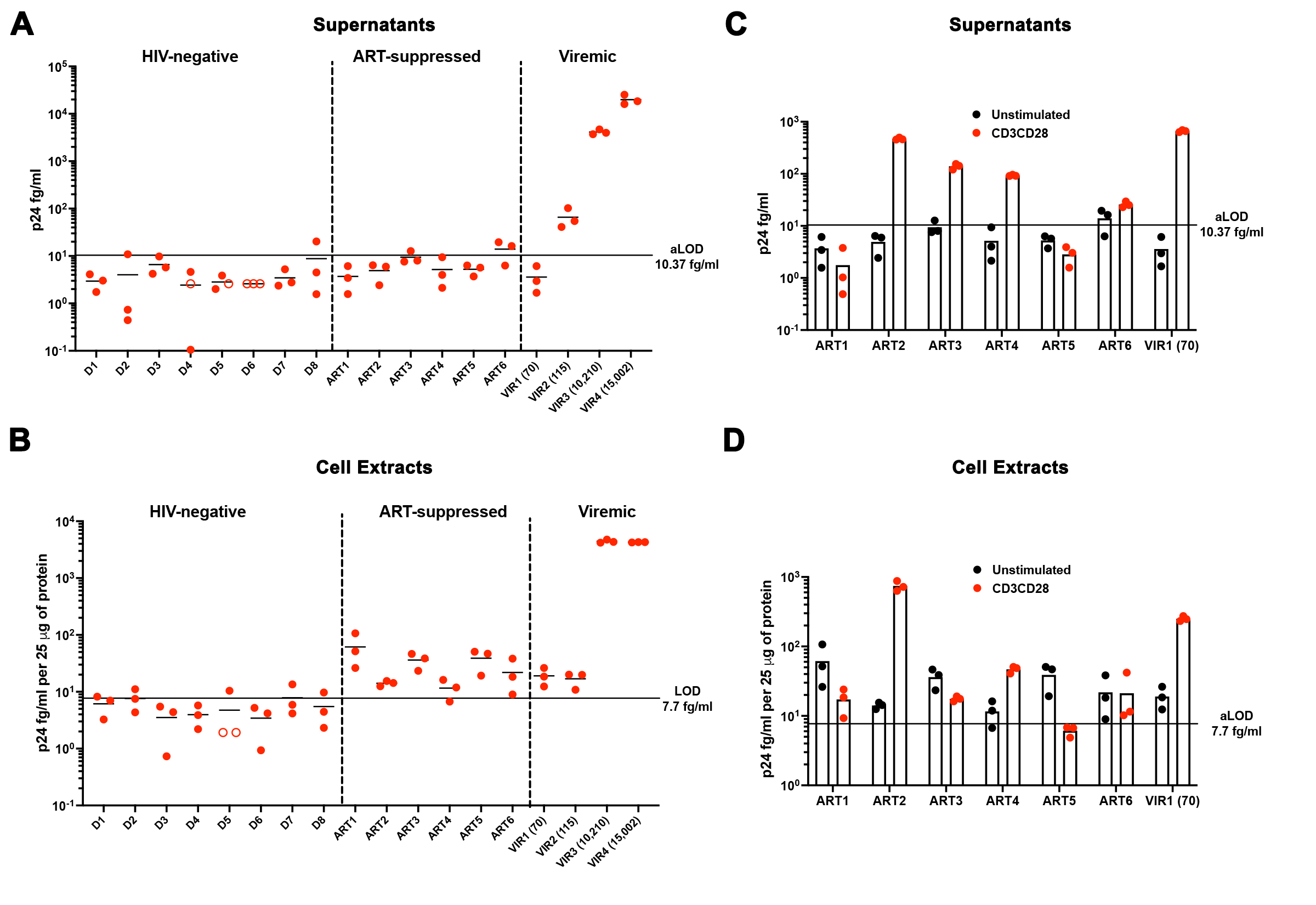


**Extended Data Figure 3. Evaluation of the homebrew Simoa planar p24 ELISA in cells isolated from PLWH.** Technical triplicates corresponding to figure 7. Levels of p24 in the supernatant **(A)** or cell lysates **(B)** of HIV-negative, ART-suppressed and viremic PLWH. Copies per ml in plasma of the viremic participants is indicated in the x-axis. Levels of p24 in the supernatant **(C)** or cell lysates **(D)** ART-suppressed PLWH upon reactivation with αCD3αCD28.


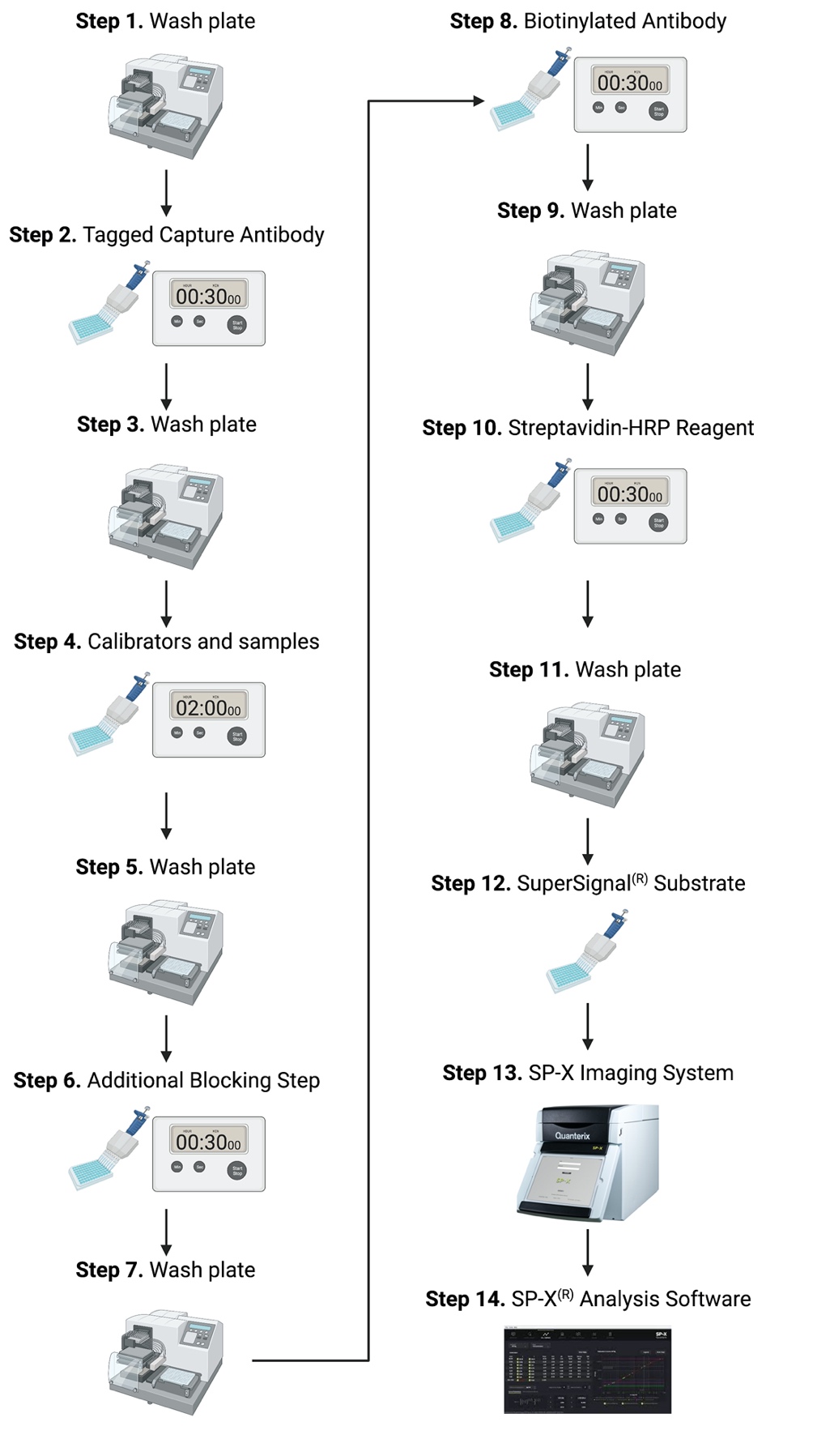


**Extended Data Figure 4. Overview of the homebrew Simoa planar p24 ELISA immunoassay protocol.** Created with Biorender.com

**Extended Table I. Assay performance in diverse matrixes.** *Optimized assay

**Extended Table II. Cost analysis of the homebrew Simoa planar p24.**
